# Supplementary material for: Subsistence transition preceded population turnover in the eastern Colombian Andes
Source: bioRxiv. 2026 Mar 25:2026.03.23.713713. Preprint. [Version 1] doi: 10.64898/2026.03.23.713713 (PMC13042078; doi:10.64898/2026.03.23.713713)
Supplement: 1 [file NIHPP2026.03.23.713713V1-supplement-1.pdf]

bioRxiv preprint doi: https://doi.org/10.64898/2026.03.23.713713; this version posted March 25, 2026. The copyright holder for this preprint (which was not certified by peer review) is the author/funder, who has granted bioRxiv a license to display the preprint in perpetuity. It is made available under aCC-BY 4.0 International license.

SUPPLEMENTAL FIGURE TITLES and LEGENDS

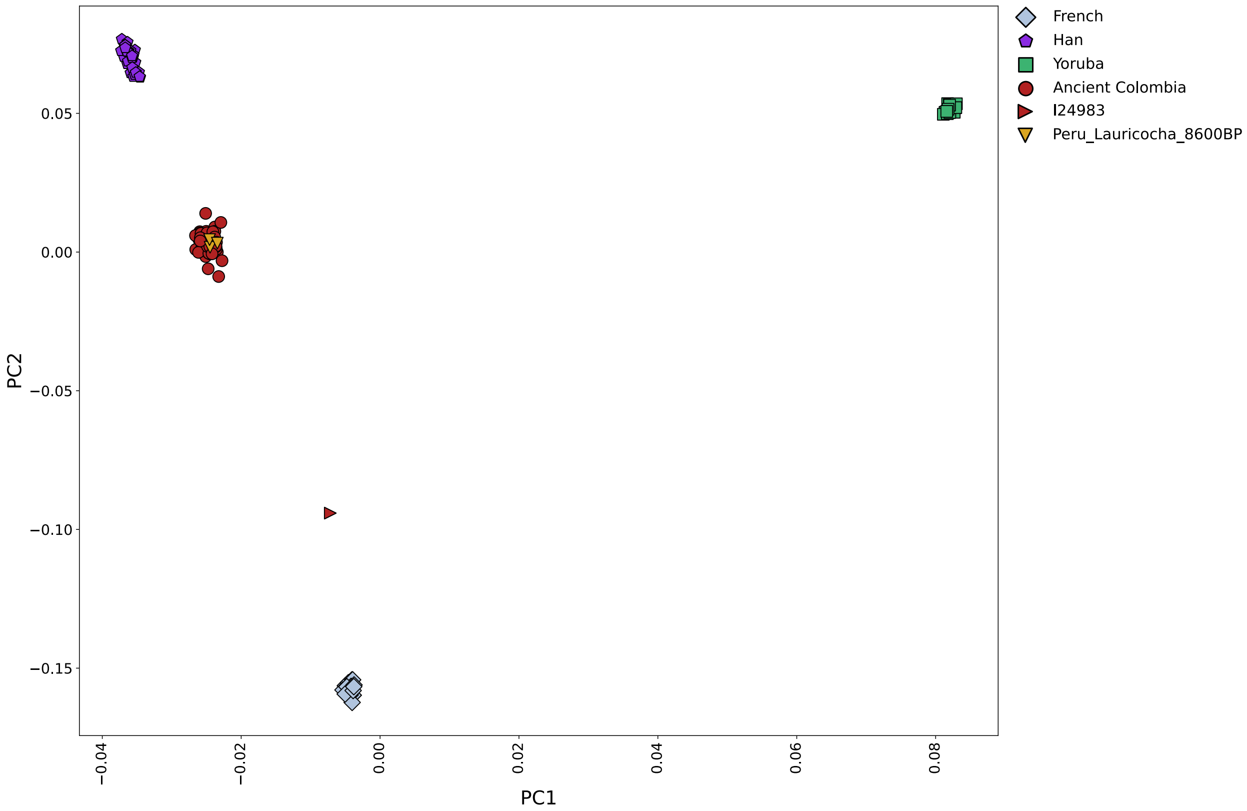

Figure S1. Worldwide PCA with eigenvectors computed using present-day Yoruba, French, and Han Chinese individuals. All ancient individuals were projected. Peru_Lauricocha_8600BP is used to represent unadmixed Native American ancestry. We excluded I24983 from all analyses based on evidence of European-related contamination.

bioRxiv preprint doi: https://doi.org/10.64898/2026.03.23.713713; this version posted March 25, 2026. The copyright holder for this preprint (which was not certified by peer review) is the author/funder, who has granted bioRxiv a license to display the preprint in perpetuity. It is made available under aCC-BY 4.0 International license.

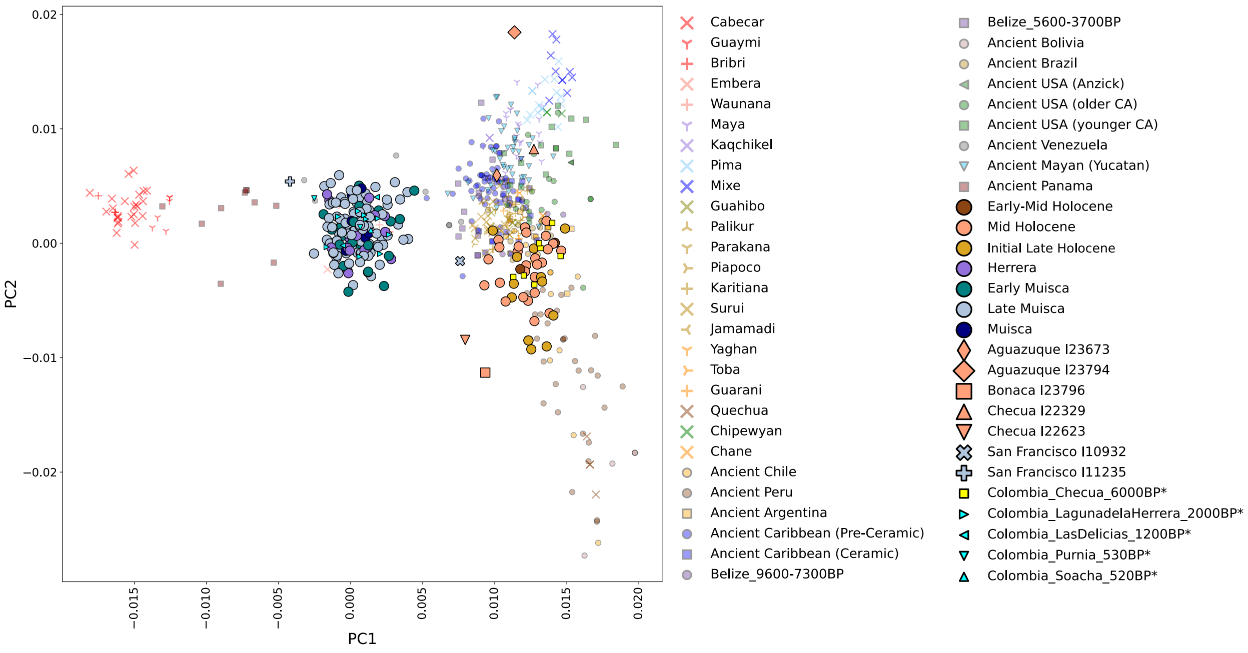

Figure S2. PCA with reference data in color. Newly reported data and published ancient Colombian data from ref. [36] represented by large and small colored symbols outlined in black. An asterisk next to a name in the legend indicates its publication in ref. [36]. A red outline denotes low coverage individuals (<15K SNPs covered on the 2M.Illumina dataset). Two PCA outlier individuals (I23673 and I10932) with >15K SNPs are labeled with diamond and X symbols, respectively.

bioRxiv preprint doi: https://doi.org/10.64898/2026.03.23.713713; this version posted March 25, 2026. The copyright holder for this preprint (which was not certified by peer review) is the author/funder, who has granted bioRxiv a license to display the preprint in perpetuity. It is made available under aCC-BY 4.0 International license.

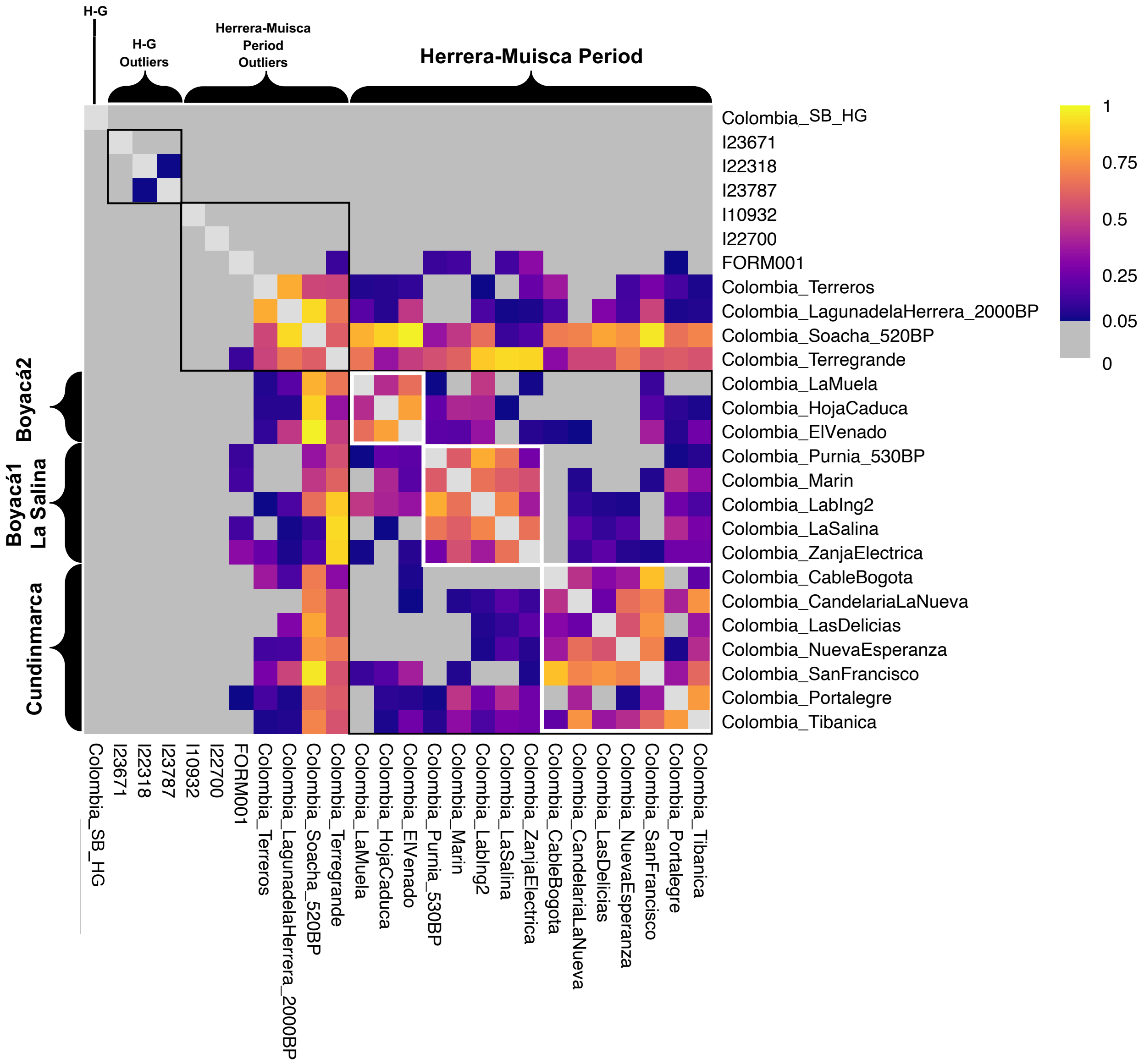

Figure S3. ***qpWave*** **heatmap illustrating genetic differences between pre-Herrera Period people with hunter-gatherer-associated ancestry (H-G) and people from Herrera-Muisca contexts and showing geographic-based genetic substructure within the Herrera-Muisca Period.** Heatmap shows pairwise *qpWave* p-values testing whether groups can be modeled as forming a clade relative to a reference set. High p-values (warm-cool colors; $p > 0.05$) support a clade-like relationship, whereas low p-values (gray; $p < 0.05$) reject a clade model. *qpWave* analyses are described in **Supplementary Information 3**.

bioRxiv preprint doi: https://doi.org/10.64898/2026.03.23.713713; this version posted March 25, 2026. The copyright holder for this preprint (which was not certified by peer review) is the author/funder, who has granted bioRxiv a license to display the preprint in perpetuity. It is made available under aCC-BY 4.0 International license.

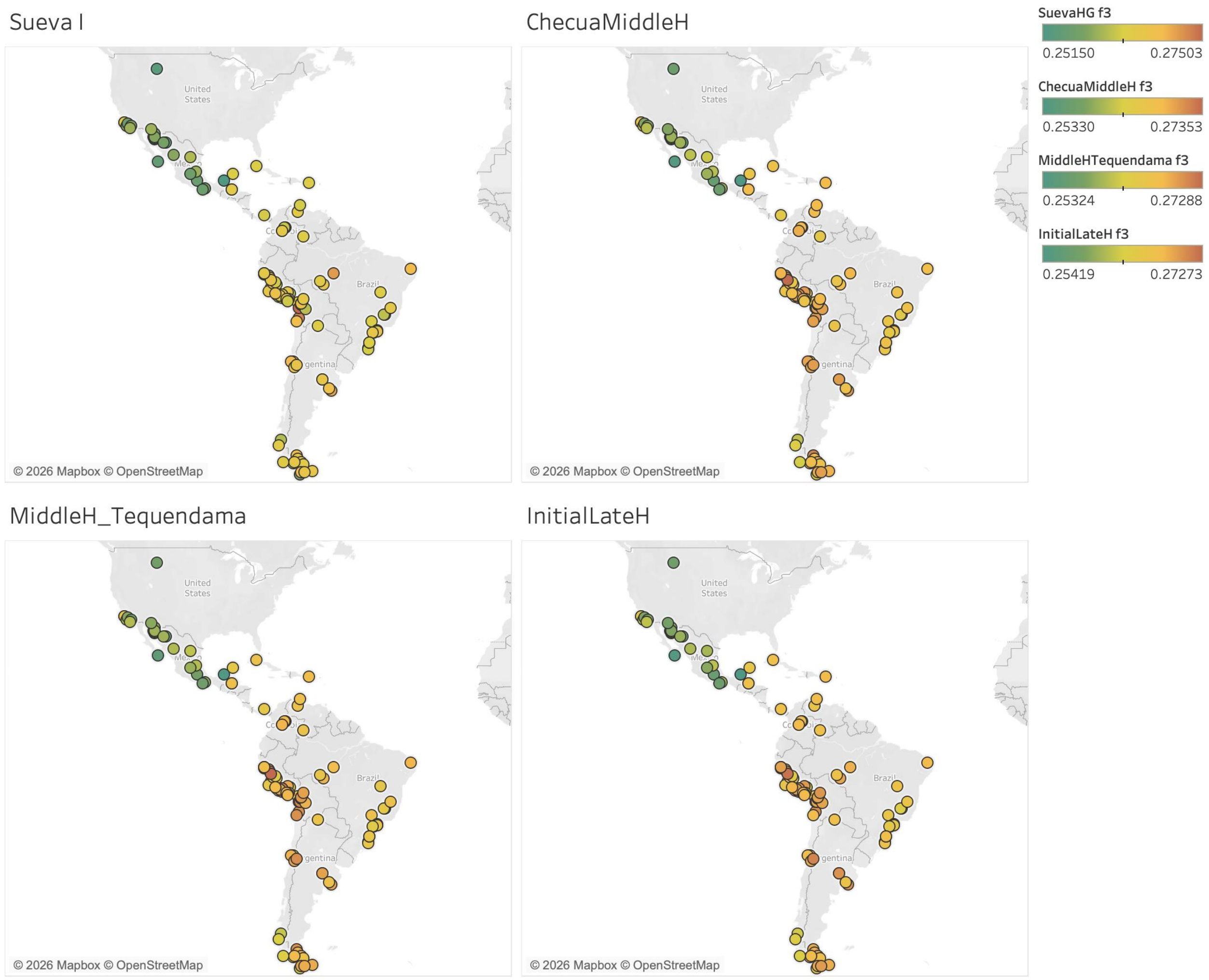

Figure S4. Outgroup-f3 heatmaps showing shared genetic drift between Native American groups and four pre-Herrera Period sub-clades: Sueva I, Checua Middle Holocene, Middle Holocene Tequendama, and Initial Late Holocene (see Figure 3). Each point represents an individual or population plotted at its geographic location (some points jittered slightly for visualization), colored by the magnitude of the f_3 value, with warmer colors indicating greater shared drift. Color scales are shown separately for each panel. Basemaps are from OpenStreetMap/Mapbox. Data are in **Table S9**.

bioRxiv preprint doi: https://doi.org/10.64898/2026.03.23.713713; this version posted March 25, 2026. The copyright holder for this preprint (which was not certified by peer review) is the author/funder, who has granted bioRxiv a license to display the preprint in perpetuity. It is made available under aCC-BY 4.0 International license.

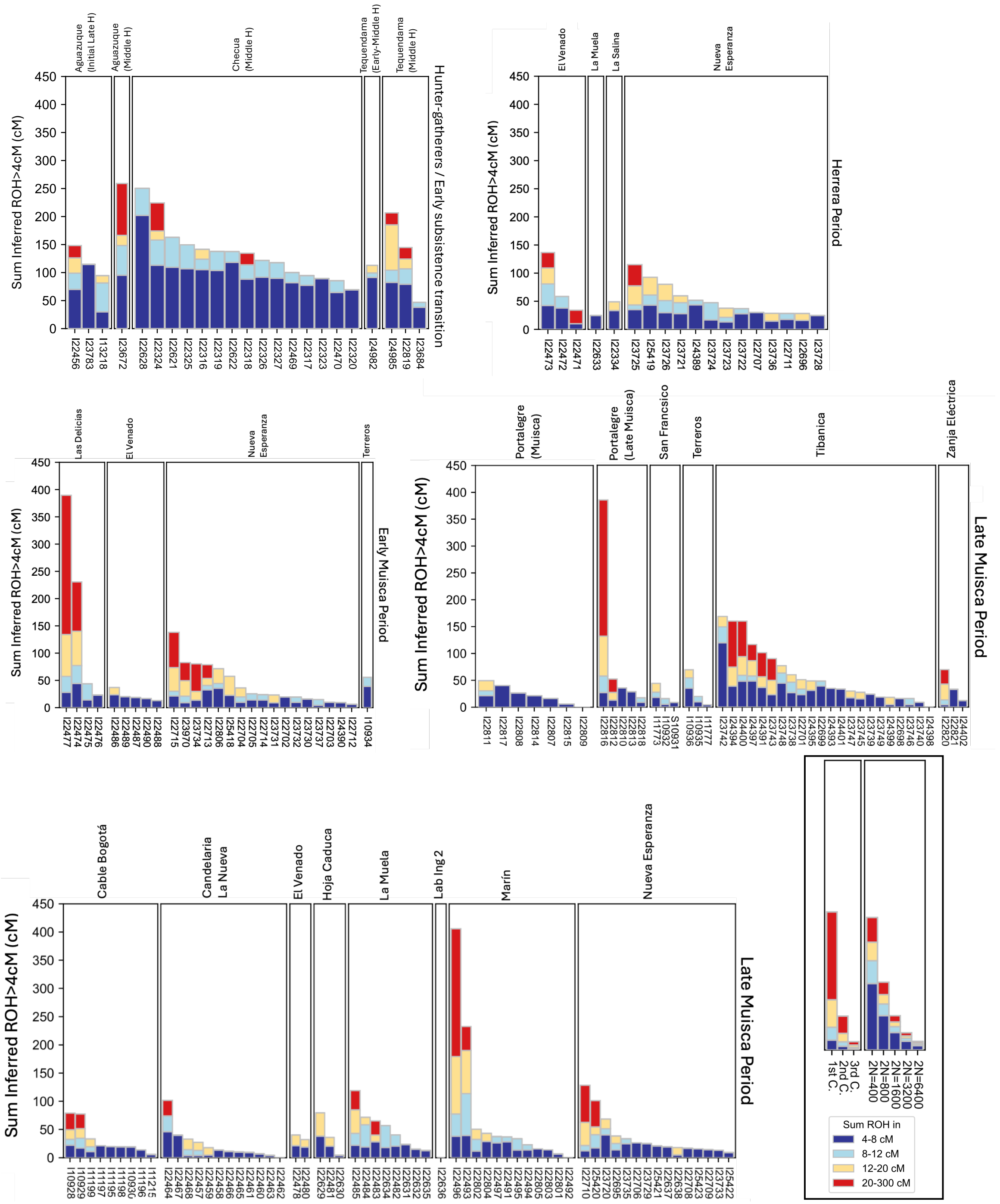

Figure S5. Figure S5. ROH calls for 173 ancient Colombian individuals. We depict inferred ROH for individuals from hunter-gatherer/early subsistence transition contexts (top left), Herrera Period contexts (top right), Early Muisca Period contexts (middle left), and Late Muisca Period contexts (middle right and bottom left). The legend (bottom right) shows analytical expectations calculated using previously-reported formulas[130] for recent parental relatedness (top right; C represents cousin) and varying population sizes (top left), as well as a color legend for four ROH length categories. Each vertical bar in this figure represents one ancient individual.

bioRxiv preprint doi: https://doi.org/10.64898/2026.03.23.713713; this version posted March 25, 2026. The copyright holder for this preprint (which was not certified by peer review) is the author/funder, who has granted bioRxiv a license to display the preprint in perpetuity. It is made available under aCC-BY 4.0 International license.

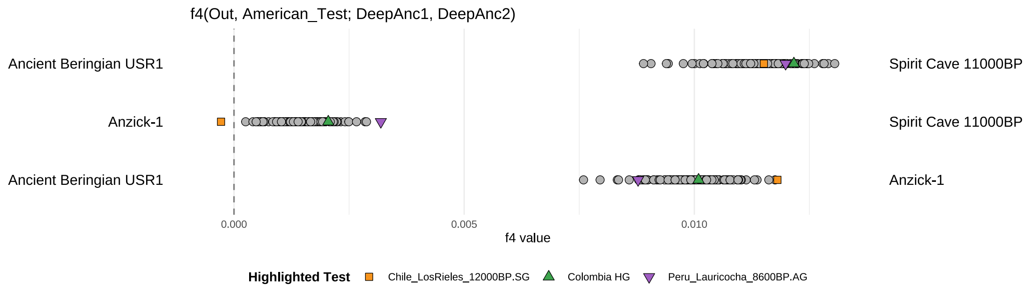

Figure S6. f_4-statistics of the form f_4(Yoruba, *NativeAmerican_Test*; *DeepAncient1*, *DeepAncient2*). Colombia_SB_HG (plotted as a green triangle) shows no evidence of ancestry related to Anzick-1 or Spirit Cave 11000BP. The statistic f_4(Yoruba, *NativeAmerican_Test*; USR1, Anzick-1/Spirit Cave 11000 BP), which measures relative allele sharing with Anzick-1 or Spirit Cave 11000BP, indicates that Colombia_SB_HG exhibits affinity to both at a level comparable to other ancient Central and South Americans. In contrast, Chile_LosRieles_12000BP.SG (orange square) shows particularly strong affinity to Anzick-1. Peru_Lauricocha_8600BP.AG (purple upside-down triangle) is a population modeled in previous work as having no Anzick-1-related ancestry and is included here for comparison. Vertical dashed line marks zero. Data are in **Table S11**.

bioRxiv preprint doi: https://doi.org/10.64898/2026.03.23.713713; this version posted March 25, 2026. The copyright holder for this preprint (which was not certified by peer review) is the author/funder, who has granted bioRxiv a license to display the preprint in perpetuity. It is made available under aCC-BY 4.0 International license.

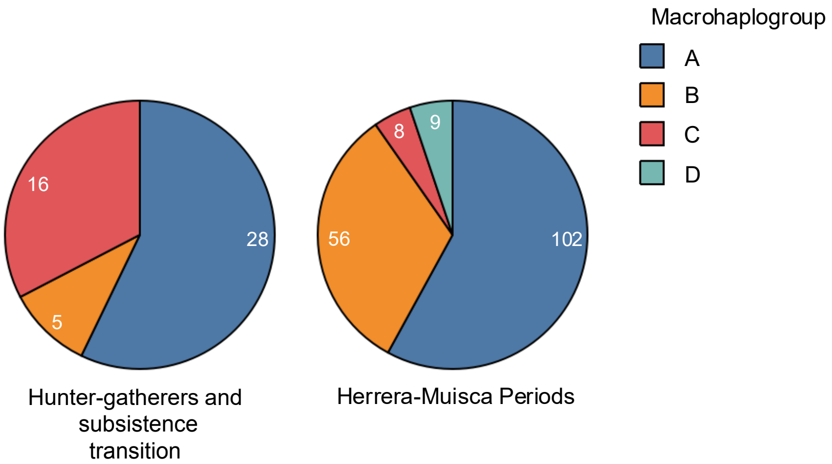

Figure S7. mtDNA macrohaplogroup composition across time on the Altiplano Cundiboyacense. Pie charts show the distribution of macrohaplogroups (A–D) among hunter-gatherers and early food producers (left) and individuals from the Herrera-Muisca Periods (right). Numbers within slices indicate the number of individuals assigned to each macrohaplogroup. The dataset includes 224 individuals in total, of which 20 were previously published[36]. Colors are consistent across panels. Data are in **Table S15**.

bioRxiv preprint doi: https://doi.org/10.64898/2026.03.23.713713; this version posted March 25, 2026. The copyright holder for this preprint (which was not certified by peer review) is the author/funder, who has granted bioRxiv a license to display the preprint in perpetuity. It is made available under aCC-BY 4.0 International license.

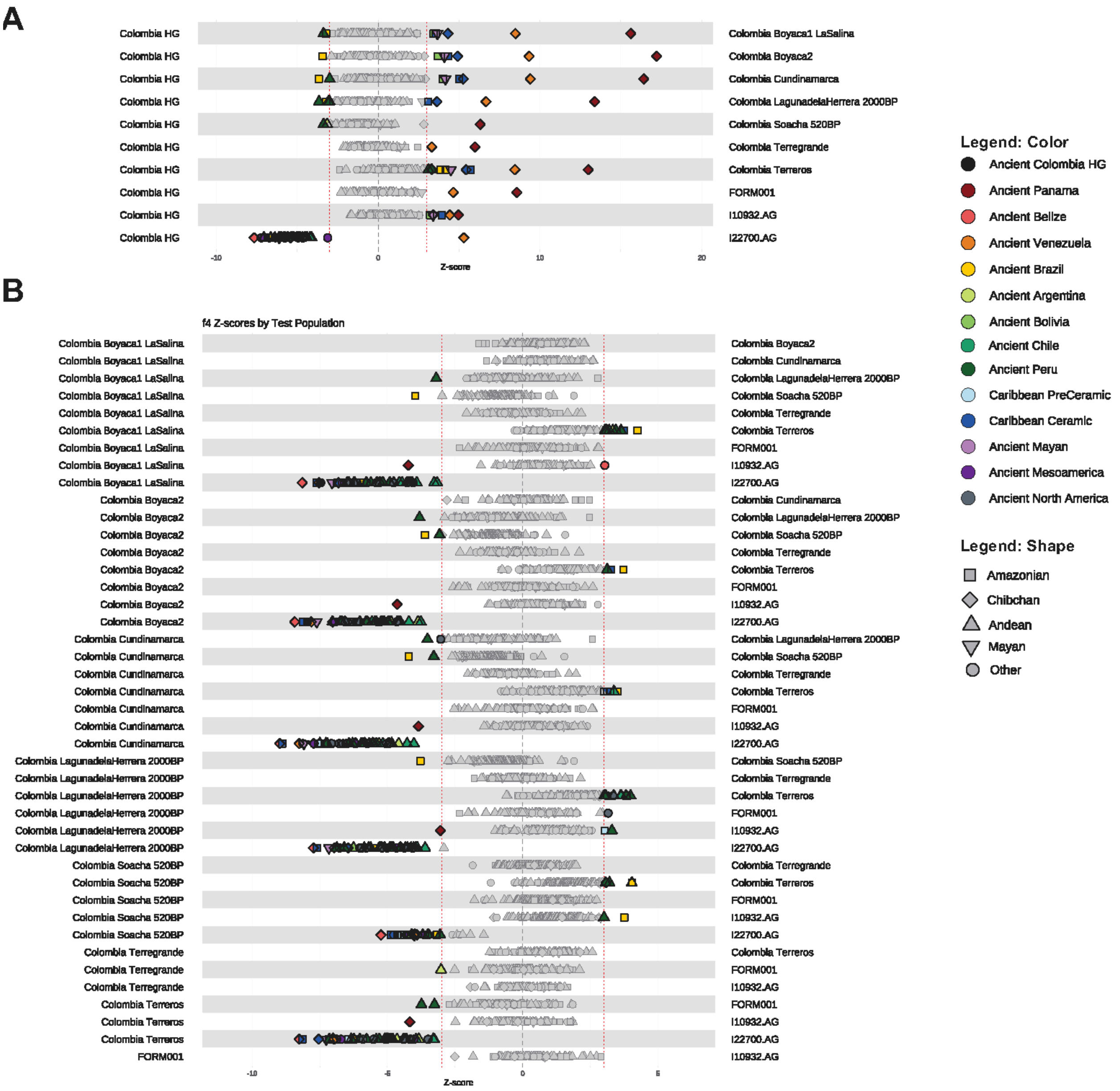

Figure S8. Differences in allele sharing patterns between the people of the Altiplano Cundiboyacense (2M dataset). A) We use the statistic f_4(Yoruba, *NativeAmerican_Test*; Colombia_SB_HG; *Colombia_HerreraMuisca*) to explore differences in allele sharing between hunter-gatherers/early subsistence transition contexts and Herrera-Muisca Period people of the Altiplano Cundiboyacense. Data are in **Table S16**. B) We use the statistic f_4(Yoruba, *NativeAmerican_Test*; *Colombia_HerreraMuisca1*; *Colombia_HerreraMuisca2*) to explore differences in allele sharing between the Herrera-Muisca Period people of the Altiplano Cundiboyacense. Data are in **Table S3**. In both panels, red dotted lines denote |Z|=3. Points associated with |Z|<3 are in gray, while those |Z|>3 are in color, with the legend on the right. The first and second affinity we used to assign the color and shape (respectively) of each *Test* population are included in the table. Only tests based on >50K SNPs are plotted.

bioRxiv preprint doi: https://doi.org/10.64898/2026.03.23.713713; this version posted March 25, 2026. The copyright holder for this preprint (which was not certified by peer review) is the author/funder, who has granted bioRxiv a license to display the preprint in perpetuity. It is made available under aCC-BY 4.0 International license.

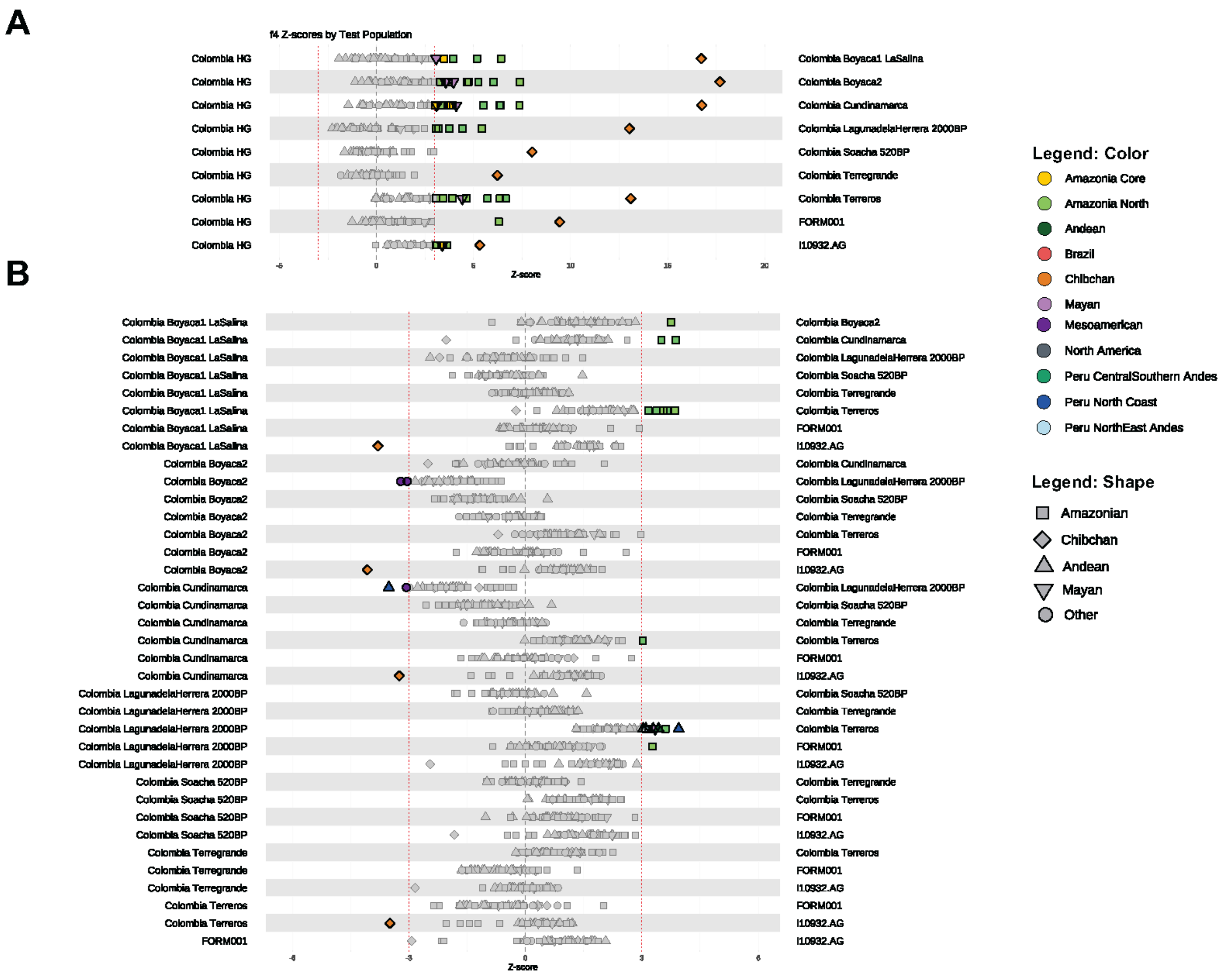

Figure S9. Differences in allele sharing patterns between the people of the Altiplano Cundiboyacense (2M.HO dataset). A) We use the statistic f_4(Yoruba, *Test_HO*; Colombia_SB_HG; *Colombia_HerreraMuisca*) to explore differences in allele sharing between hunter-gatherers/early subsistence transition contexts and Herrera-Muisca Period people of the Altiplano Cundiboyacense. Data are in **Table S17**. B) We use the statistic f_4(Yoruba, *Test_HO*; *Colombia_HerreraMuisca1*; *Colombia_HerreraMuisca2*) to explore differences in allele sharing between Herrera-Muisca Period people of the Altiplano Cundiboyacense. Data are in **Table S4**. In both panels, red dotted lines denote |Z|=3. Points associated with |Z|<3 are in gray, while those |Z|>3 are in color, with the legend on the right. The first and second affinity we used to assign the color and shape (respectively) of each *Test* population are included in the table. Only tests based on >50K SNPs are plotted. There were no tests based on >50K SNPs for I22700.

bioRxiv preprint doi: https://doi.org/10.64898/2026.03.23.713713; this version posted March 25, 2026. The copyright holder for this preprint (which was not certified by peer review) is the author/funder, who has granted bioRxiv a license to display the preprint in perpetuity. It is made available under aCC-BY 4.0 International license.

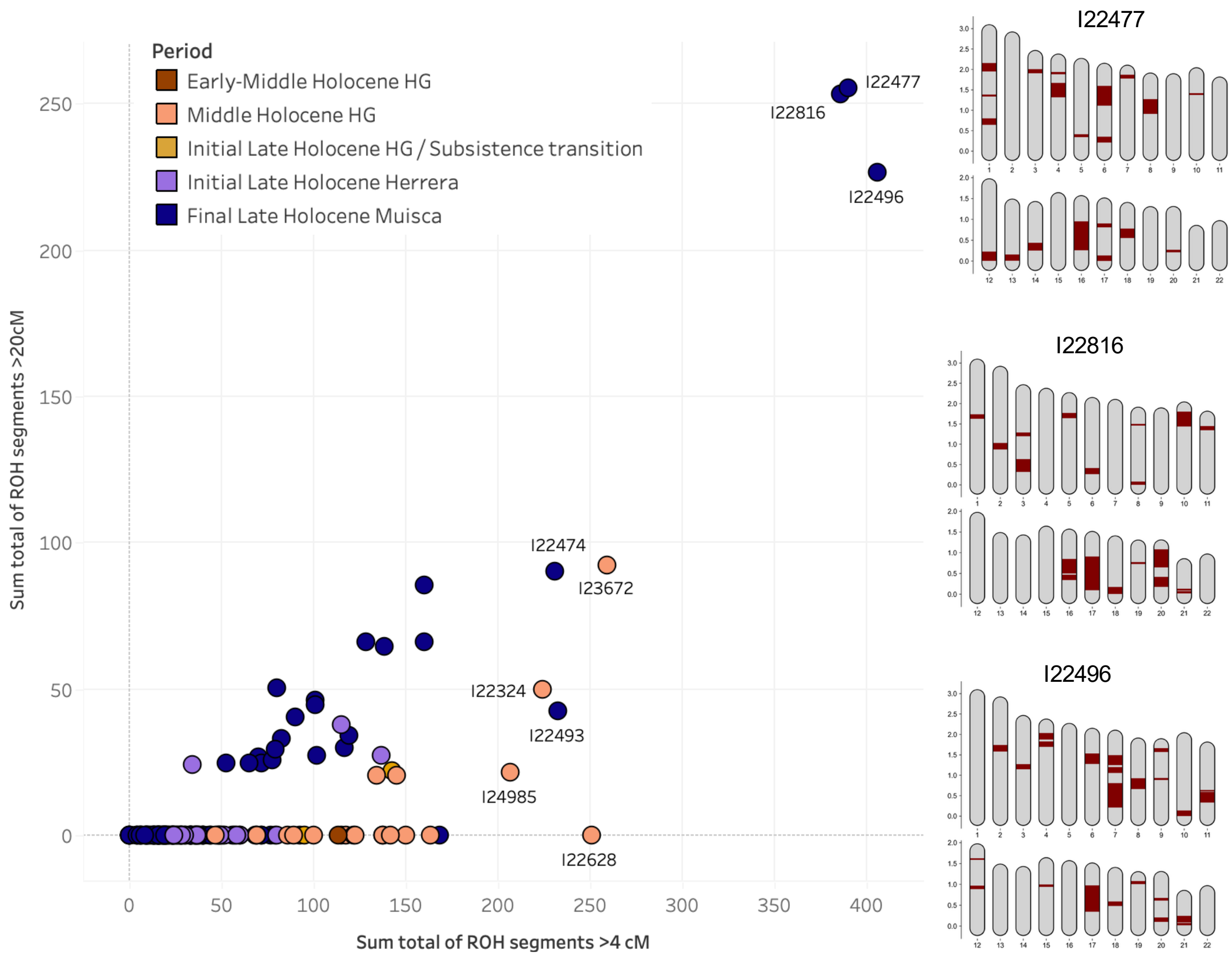

Figure S10. Runs of homozygosity (ROH) across individuals from the Altiplano Cundiboyacense. Left: plot of sum total of sum total of ROH segments >4cM (x-axis) against sum total of ROH segments >20cM (y-axis) for 173 individuals with sufficient coverage (at least 300,000 SNPs overlapping a set of 2M target SNPs). Each point represents one individual, color coded by time period. Nine individuals with >200cM ROH >4cM are labeled. Data are in **Table S10**. Right: karyotypes for three Muisca Period individuals with very high levels of ROH >20cM, indicating consanguinity. Gray outlines represent autosomes and red shaded sections indicate ROH segments.

bioRxiv preprint doi: https://doi.org/10.64898/2026.03.23.713713; this version posted March 25, 2026. The copyright holder for this preprint (which was not certified by peer review) is the author/funder, who has granted bioRxiv a license to display the preprint in perpetuity. It is made available under aCC-BY 4.0 International license.

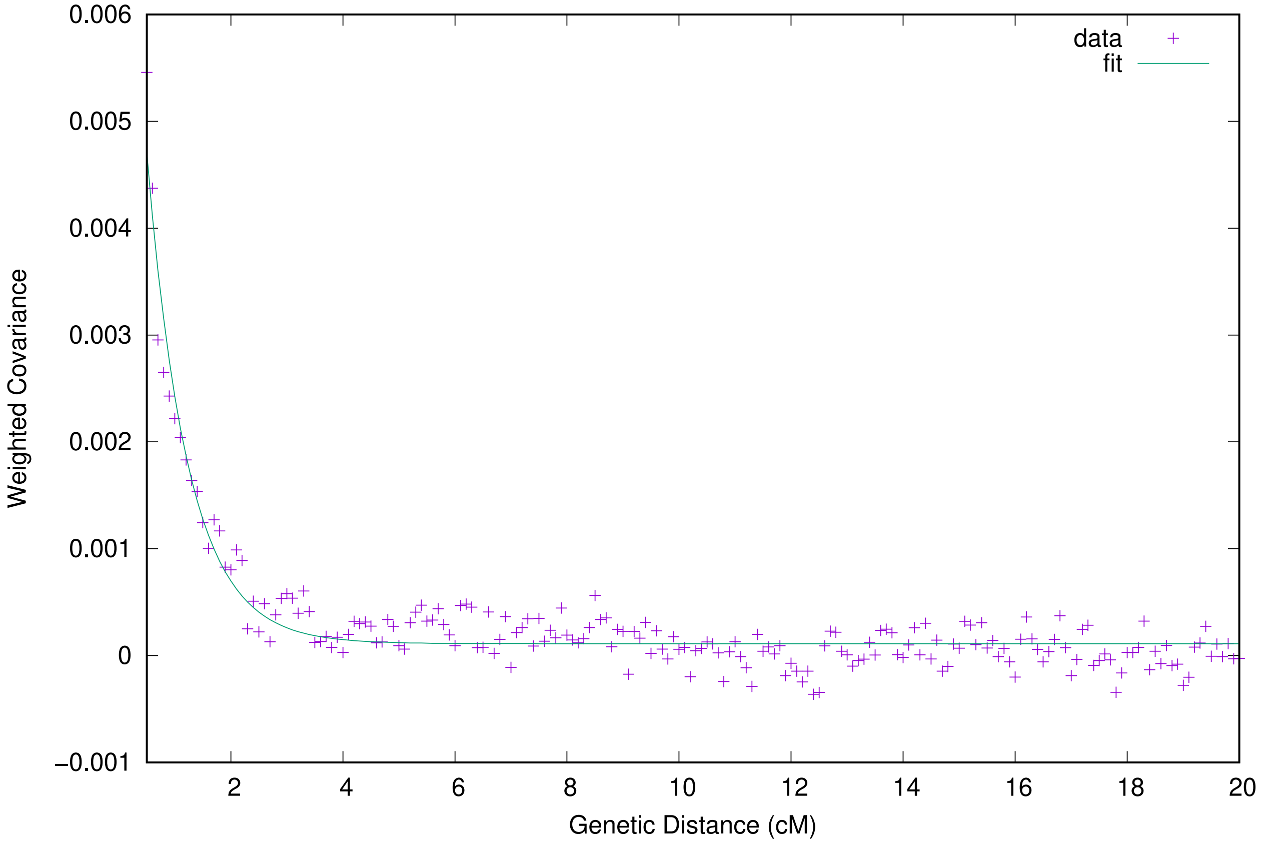

Figure S11. Ancestry covariance decay curve for Herrera-Muisca Period people (target) inferred using a set of pooled ancient and modern Chibchan-related individuals as one reference group and Amazonian-related individuals as a second reference group (composition of groups detailed in Table S25). Weighted ancestry covariance is shown as a function of genetic distance (cM). Purple crosses represent the observed data, and the solid line shows the best-fitting exponential decay model used by DATES to infer admixture timing. The average date of admixture is estimated at 137 ± 15 generations before the target individuals lived. We observe a strong ancestry covariance decay consistent with admixture, with high statistical support (Z = 8.9).
